# CCR5 receptor antagonism inhibits hepatitis C virus (HCV) replication *in vitro*

**DOI:** 10.1371/journal.pone.0224523

**Published:** 2019-10-29

**Authors:** Jason T. Blackard, Ling Kong, Susan D. Rouster, Rebekah Karns, Paul S. Horn, Shyam Kottilil, M. Tarek Shata, Kenneth E. Sherman

**Affiliations:** 1 Division of Digestive Diseases, Department of Internal Medicine, University of Cincinnati College of Medicine, Cincinnati, OH, United States of America; 2 Digestive Health Center, Cincinnati Children’s Hospital, Cincinnati, OH, United States of America; 3 Department of Pediatrics, University of Cincinnati College of Medicine, Cincinnati, OH, United States of America; 4 Neurology Division, Cincinnati Children’s Hospital Medical Center, Cincinnati, OH, United States of America; 5 University of Maryland, Baltimore, MD, United States of America; Saint Louis University, UNITED STATES

## Abstract

**Background and aim:**

The hepatitis C virus (HCV) is a single-strand RNA virus that infects millions of people worldwide. Recent advances in therapy have led to viral cure using two- and three- drug combinations of direct acting inhibitors of viral replication. CCR5 is a chemokine receptor that is expressed on hepatocytes and represents a key co-receptor for HIV. We evaluated the effect of CCR5 blockade or knockdown on HCV replication in Huh7.5_JFH1_ cells.

**Methods:**

Cells were exposed to varying concentrations of maraviroc (CCR5 inhibitor), cenicriviroc (CCR2/CCR5 inhibitor), sofosbuvir (nucleotide polymerase inhibitor), or raltegravir (HIV integrase inhibitor).

**Results:**

HCV RNA was detected utilizing two qualitative strand-specific RT-PCR assays. HCV core antigen and NS3 protein was quantified in the supernatant and cell lysate, respectively. siRNA was utilized to knockdown CCR5 gene expression in hepatocytes. Alternatively, anti-CCR5 antibodies were employed to block the receptor. Supernatant levels of HCV RNA (expressed as fold change) were not reduced in the presence of raltegravir but were reduced 8.55-fold and 12.42-fold with cenicriviroc and maraviroc, respectively. Sofosbuvir resulted in a 16.20-fold change in HCV RNA levels. HCV core and NS3 protein production was also reduced in a dose-dependent manner. Two distinct anti-CCR5 antibodies also resulted in a significant reduction in HCV protein expression, as did siRNA knockdown of CCR5 gene expression.

**Conclusions:**

These data provide evidence that CCR5 modulation could have a significant effect on HCV replication in an *in vitro* system. Further evaluation of the role of CCR5 inhibition in clinical settings may be warranted.

## Introduction

It is well established that HIV enters target cells by forming a complex consisting of the viral envelope glycoprotein (trimeric gp120), CD4 receptor, and members of the chemokine co-receptor family. A variety of chemokine receptors may serve as HIV entry cofactors, with CCR5 and CXCR4 being the most common. CCR5 is the major co-receptor for macrophage tropic (M-tropic) HIV isolates, while CXCR4 is the primary co-receptor utilized by T cell tropic (T-tropic) HIV isolates.[1] Binding of HIV virions or soluble gp120 to their receptors triggers a broad spectrum of signaling pathways that modulates the activation state of target cells. The CCR5 delta-32 mutation reduces or prevents infection with M-tropic HIV and limits AIDS progression in heterozygotic carriers.[2, 3]

The relationship of CCR5 to other viral infections such as HCV is less clear. HCV replicates primarily in hepatocytes, although some extrahepatic cell types may demonstrate limited permissiveness.[4] CCR5 is expressed on hepatocytes, as well as stellate cells.[5, 6] CCR5 has been implicated in HCV susceptibility, hepatic injury, and response to therapy.[7] Though some studies suggested that the heterozygotic CCR5 delta-32 mutation could be associated with alterations in HCV RNA levels among infected individuals, this effect was not observed in a well-controlled epidemiologic analysis reported by Wasmuth *et al*.[8] However, HCV-specific immune responses may be impaired by the CCR5 delta-32 mutation as well.[9] Wald *et al*. suggested that the CCR5 delta-32 allele may be associated with decreased hepatic inflammation in HCV-infected patients using histopathologic outcome measures.[10] Intervention with agents such as maraviroc that block CCR5 have been associated with decreased hepatic fibrosis in HCV-infected patients; however, the change in HCV viral load was not statistically significant.[11] Cenicriviroc–a dual CCR2/CCR5 inhibitor–is under active investigation to evaluate its modulation of hepatic fibrosis in patients with non-alcoholic steatohepatitis (NASH) and has previously been shown to inhibit HIV in the context of combination antiretroviral regimen.[12, 13]

We previously demonstrated that CCR5 blockade or mutation is associated with decreased hepatic fibrosis among patients with HIV infection, including those with HCV coinfection.[14] In the current study, we explored the effects of CCR5 inhibition or knockdown on HCV replication in tissue culture-based model systems to clarify the observed associations between CCR5 and HCV replication.

## Materials and methods

### Cell culture and drug/antibody inhibition studies

The human hepatocyte cell line Huh7.5 was provided by Apath LLC (St. Louis, MO) and maintained in Dulbecco’s Modified Eagle’s Medium (DMEM) high glucose supplemented with 10% fetal bovine serum (FBS), penicillin (100 U/mL), streptomycin (100 mg/mL), and non-essential amino acids. The Huh7.5_JFH1_ cell line–which releases infectious genotype 2a virions into the cell culture supernatant–was provided by Dr. Guangxiang Luo, (Cai, et al. [15]) and maintained in DMEM with 10% FBS and 5 ug/mL of blasticidin.

1 x 10^5^ Huh7.5_JFH1_ cells were plated in 24-well format. After 24 hours, cells were incubated with 0.0025, 0.25, or 25 ug/mL of cenicriviroc (CVC), maraviroc, raltegravir, or sofosbuvir. CVC (Tobira; South San Francisco, CA) is a dual inhibitor of the CCR2 and CCR5 pathways. Maraviroc (Pzifer Inc.; New York, NY) is an HIV entry inhibitor that binds the CCR5 receptor. Raltegravir (Merck; Kenilworth, NJ) is an HIV integrase inhibitor that blocks the integration of linear HIV DNA into the host cell chromosome. Sofosbuvir (Gilead; Foster City, CA) is an inhibitor of the HCV RNA-dependent RNA polymerase coded for by the viral NS5B gene.

For studies using antibodies, 1 x 10^5^ Huh7.5_JFH1_ cells were plated in 24-well format. After 24 hours, cells were incubated with a 1:100 dilution of the following antibodies from the NIH AIDS Reagent Program Division of AIDS, NIAID, NIH: anti-CXCR4 (NIH 4083), anti-CCR3 (NIH 4923), or anti-CCR5 (NIH 3933) antibodies. IgG_1_ isotype control antibody (IC002A) was obtained from R&D Systems (Minneapolis, MN).

The chemokines MIP-1 alpha MIP-1 beta, and RANTES/CCL5 were quantified in cell supernatants at day 1 and day 3 after addition of CVC, maraviroc, raltegravir, or sofosbuvir. Immune markers were quantified by analyte-specific bead-based Luminex multiplex immunoassays (EMD Millipore Corporation). Mean fluorescence intensity for the analytes was detected on a flow-based Luminex platform, and concentrations were calculated based on a standard curve of the reference with a known concentration supplied by the manufacturer.

### Evaluation of HCV replication

RNA from cell lysates was extracted using TRIzol (Invitrogen; Carlsbad, CA), washed, and resuspended in 50 uL of DEPC-treated dH_2_O. RNA from 140 uL of culture supernatant was extracted using the QIAamp Viral RNA Kit (Qiagen; Valencia, CA), and eluted in 60 uL of DEPC-treated dH_2_O. HCV RNA was detected utilizing two qualitative strand-specific RT-PCR assays as described previously.[16, 17] The sensitivity of the HCV PCR was previously determined to be approximately 230 copies HCV/uL. PCR primers included the HCV-II sense primer (5’–CAC TCC CCT GTG AGG AAC T– 3’, nucleotides [nt] 38–56 of the 5′ UTR) and the HCV-I antisense primer (5’–TGG ATG CAC GGT CTA CGA GAC CTC– 3’, nt 342–320) or the antisense primer KY78 (5’–CTC GCA AGC ACC CTA TCA GGC AGT– 3’, nt 311–288) and sense primer KY80 (5’–GCA GAA AGC GTC TAG CCA TGG CGT– 3’, nt 68–91). 30 cycles of PCR (94°C for 30 seconds, 58°C for 1 minute, and 72°C for 2 minutes) were performed, and PCR products (295 base pairs in length for HCV-I/-II and 244 base pairs for KY78/80) were visualized by gel electrophoresis. HCV core protein was quantified in cell culture supernatants by the QuikTiter HCV Core Antigen ELISA Kit (Cell Biolabs, Inc.; San Diego, CA) with a lower limit of detection of 1 ng/mL. HCV NS3 was quantified in cell lysates using the Quantitative HCV NS3 ELISA Kit (BioFront Technologies Inc.; Tallahassee, FL) with a lower limit of detection of 9 ng/mL.

### CCR5 knockdown in hepatocytes

The Huh7.5 cell line was incubated with shRNA sequence corresponding to CCR5 (GGA AAT ACA ATG TGT CAA CTC; location 411) or scramble (GeneCopoeia, Inc.; Rockville, MD) at a concentration of 1 ug per 1,000,000 cells in 6-well plate format for 3 weeks in the presence of 100 ug hygromycin /mL to generate the Huh7.5_shCCR5_ or Huh7.5_shSCRAMBLE_ cell lines. CCR5 receptor expression was then evaluated using the CCR5 Cell-Based ELISA Kit (KA2647) from Abnova (Taipei City, Taiwan). 1 x 10^5^ Huh7.5_shCCR5_ or Huh7.5_shSCRAMBLE_ were plated in 24-well format and infected with infectious virions harvested from the Huh7.5_JFH1_ cell at a concentration of 0.5 TCID_50_. HCV replication was quantified as described above.

### RNAseq analysis

The NEBNext Poly (A) mRNA Magnetic Isolation Module (New England Biolabs, Ipswich, MA) was utilized for polyA RNA purification with a total of 300 ng good quality total RNA as input. A PrepX mRNA Library kit (WaferGen) combined with Apollo 324 NGS automated library prep system was used for library preparation, which is an RNA ligation-based method to maintain strand specificity. Sample-specific indices were added to each adaptor-ligated cDNA sample, using the universal and index-specific primer with a limited PCR cycle number, and the amplified library was cleaned by AMPure XP beads in the Apollo 324 system with a final elution volume of 16 μl. To confirm the quality and yield of the purified library, 1 μl was analyzed by Bioanalyzer (Agilent, Santa Clara, CA) using a DNA high sensitivity chip. To accurately quantify the library concentration for the clustering, the library was diluted 1:10^4^ in dilution buffer (10 mM Tris-HCl, pH 8.0 with 0.05% Tween 20), and qPCR measured by Kapa Library Quantification kit (Kapabiosystem, Woburn, MA) using ABI's 9700HT real-time PCR system (Thermo Fisher). Individually indexed and compatible libraries were proportionally pooled (~25 million reads per sample in general) for clustering in cBot system (Illumina, San Diego, CA). Libraries at a final concentration of 15 pM were clustered onto a single read flow cell using Illumina TruSeq SR Cluster kit v3, and sequenced to 50 bp using TruSeq SBS kit on Illumina HiSeq system.

Following removal of primers and barcodes, raw reads were aligned to the Hg38 genome with annotations provided by UCSC, with output of transcripts per million. Data were normalized with a 75^th^ percentile shift and baselined to the median of all samples. Analyses were constrained to reasonably-expressed transcripts (n = 8417). Cenicriviroc and maraviroc triplicate samples from day 1 post-drug incubation were compared to day 1 samples treated with sofosbuvir, with significance defined as p < 0.05 and fold-change > 1.5. We further compared day 1 sofosbuvir-treated to untreated samples, and removed those genes from the cenicriviroc versus sofosbuvir and maraviroc versus sofosbuvir gene sets to identify those genes specific to the experimental treatments rather than the antiviral activity of sofosbuvir. Cenicriviroc and maraviroc-specific gene sets were submitted to toppcluster (toppcluster.cchcm.org) and cytoscape for ontological analysis and network generation. All transcriptomic analyses were performed in GeneSpring GX 14.9.

### Statistical analysis

Data are expressed as mean ± SEM. Means were tested for statistical significance using the Student’s t-test linear models, and non-parametric methods as indicated. Linear mixed models were based on one- and two-way ANOVAs with replicate as a random effect. A significance level of p < 0.05 was applied when comparing virus-treated with untreated cells. No adjustments were made for multiple testing. Statistical analyses were performed using GraphPad Prism 5 (San Diego, CA) and SAS® version 9.4 (SAS Institute Inc., Cary, NC).

## Results

**Fig 1:**
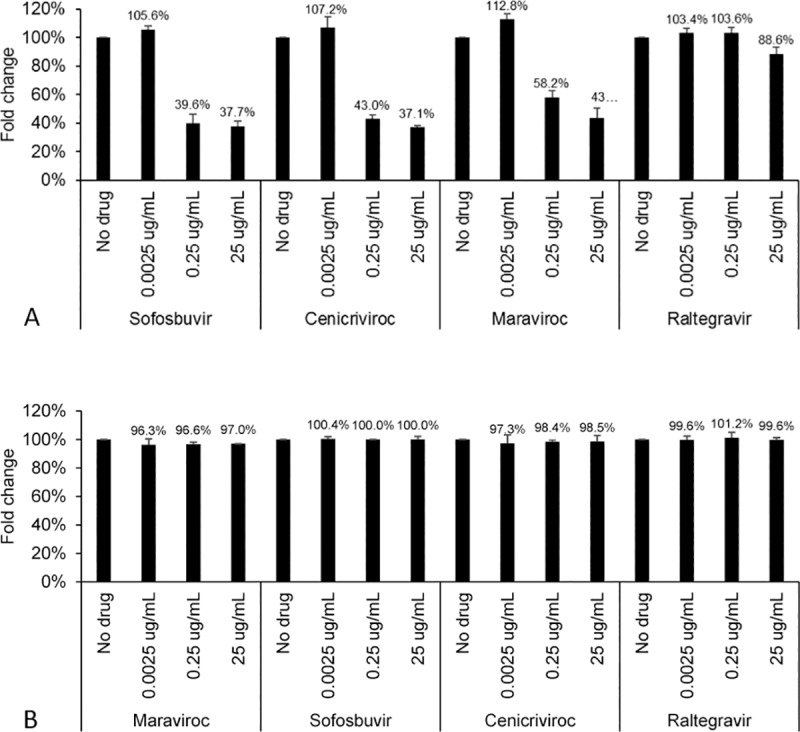
Antiviral effects on Huh7.5_JFH1_ cells. Cells were exposed to 0–25 ug/mL of sofosbuvir, cenicriviroc, maraviroc, or raltegravir for 24 hours. **(A)** HCV core protein production was quantified by ELISA in supernatants at day 1 post-incubation. Using replicates as a blocking variable and combining drug/dose by fold-change, overall mean score difference based upon rank scores using Cochran-Mantel-Haenszel statistic, p = 0.038. Two-way ANOVA using replicate as block variable, demonstrates significant differences by drug, dose, and drug x dose (p < 0.001). **(B)** Cell number was determined for each experimental condition. No significant differences by drug or dose were observed.

It is well known the CCR5 antagonists suppress HIV replication. However, their impact on other common co-infections such as HCV is unknown. The Huh7.5_JFH1_ cell line, which expresses infectious HCV, was exposed to 3 different doses of cenicriviroc or maraviroc as CCR5 antagonists, as well as sofosbuvir and raltegravir. As shown in Fig 1A, HCV core protein levels were significantly reduced in the presence of 0.25 and 25 ug/mL of cenicriviroc and maraviroc, although there was no effect with raltegravir. As expected, sofosbuvir reduced HCV core protein levels significantly. Cell number was not impacted by the individual antiviral agent nor its dose (Fig 1B).

**Fig 2:**
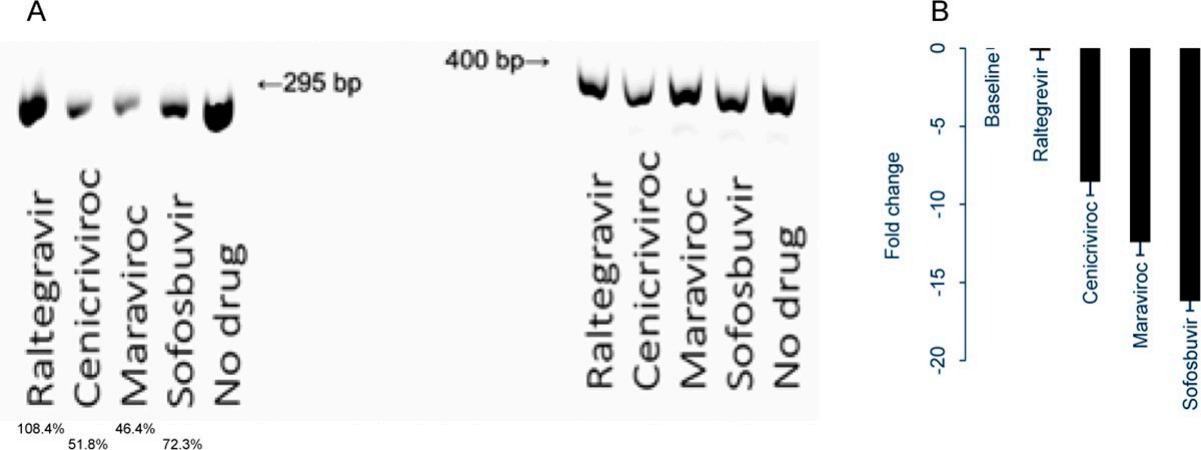
HCV RNA detection after antiviral exposure. Huh7.5_JFH1_ cells were exposed to 0–25 ug/mL of sofosbuvir, cenicriviroc, maraviroc, or raltegravir for 24 hours. **(A)** HCV negative-strand RNA (left) and GAPDH (right) were detected by qualitative RT-PCR in cell lysates, and **(B)** HCV positive-strand RNA in supernatant was expressed as reduction (fold change) over 24 hours.

The impact of CCR5 antagonists was evaluated further by qualitative HCV RNA expression. While all antivirals evaluated–cenicriviroc, maraviroc, raltegravir, and sofosbuvir–had no impact on expression of the cellular housekeeping gene GAPDH, cellular expression of HCV negative-sense RNA was reduced in the presence of cenicriviroc and maraviroc (Fig 2A). As expected, addition of sofosbuvir also resulted in decreased HCV negative-sense RNA expression. Using a quantitative assay, supernatant levels of HCV RNA (expressed as fold change) were reduced minimally (0.15) in the presence of raltegravir but 8.55 and 12.42 in the presence of cenicriviroc and maraviroc, respectively (Fig 2B). Sofosbuvir resulted in a 16.20-fold change in HCV RNA level.

The chemokines MIP-1 alpha, MIP-1 beta, and RANTES/CCL5 were quantified in the cell supernatants after addition of CVC, maraviroc, raltegravir, or sofosbuvir. RANTES was not detected or was detected near the lower limit of assay detection for all conditions (data not shown). As shown in S1 Fig, MIP-1 alpha levels were largely unchanged at day 1 post-antiviral treatment. At day 3, MIP-1 alpha levels were elevated in all conditions and similar with the exception of lower MIP-1 alpha levels in the presence of sofosbuvir. MIP-1 beta expression at day 1 was somewhat lower in the presence of cenicriviroc and maraviroc–but not sofosbuvir–compared to the no-drug control condition. No MIP-1 beta was detected in the presence of raltegravir. By day 3 post-treatment, MIP-1 beta levels were elevated in all conditions and similar with the exception of lower levels in the presence of sofosbuvir.

**Fig 3:**
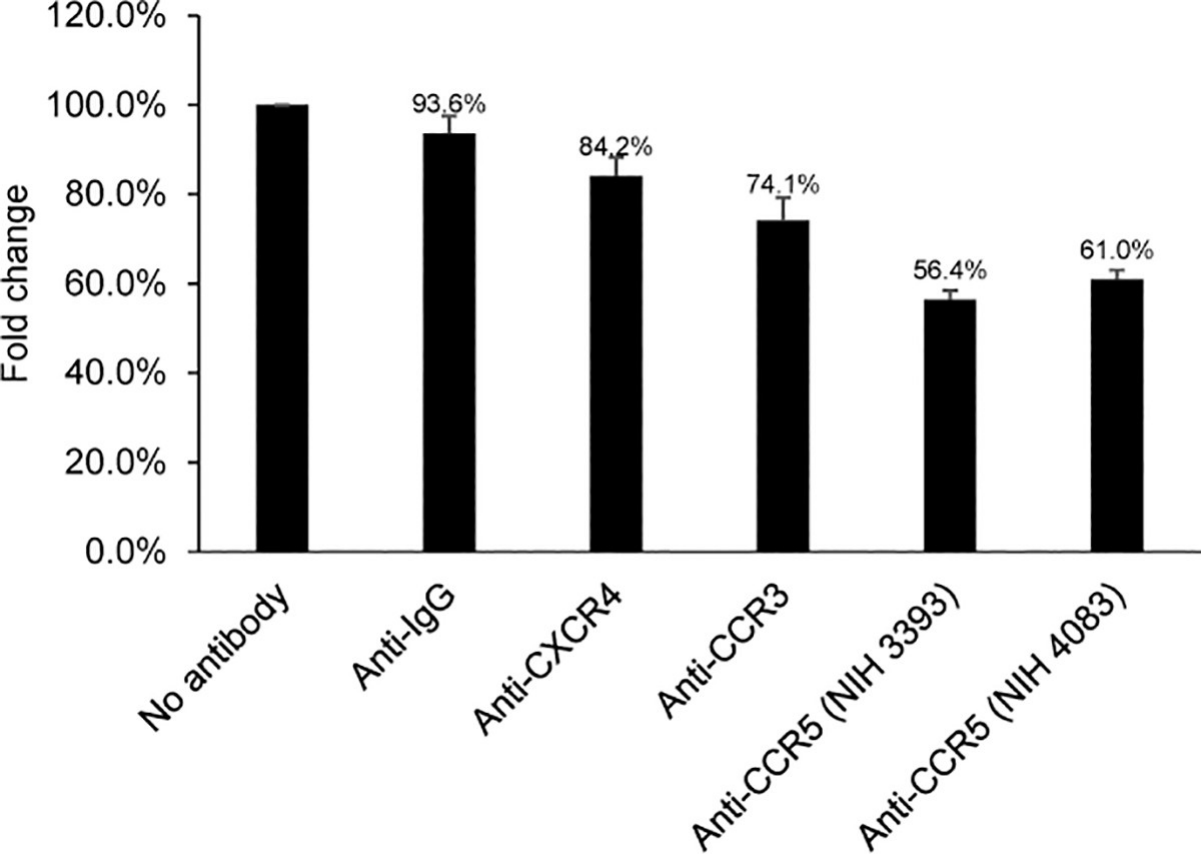
HCV core protein production after antibody treatment. Huh7.5_JFH1_ cells were exposed to a 1:100 dilution of anti-IgG (RD IC002A), anti-CXCR4 (NIH 4083), anti-CCR3 (NIH 4923), anti-CCR5 (NIH 3933), or anti-CCR5 (NIH 4083). HCV core protein production was quantified by ELISA in supernatants at day 1 post-incubation. All comparisons to anti-IgG significant (p < 0.05) using comparison of least square means based on a one-way ANOVA.

CCR5 antagonism was also explored in the presence of anti-CCR5 antibodies as shown in Fig 3. HCV core protein levels were similar in the presence of anti-IgG_1_ and anti-CXCR4 antibodies compared to the no antibody (background) condition. However, the addition of either of two distinct anti-CCR5 antibodies resulted in a significant (p < 0.05) reduction in HCV protein expression.

**Fig 4:**
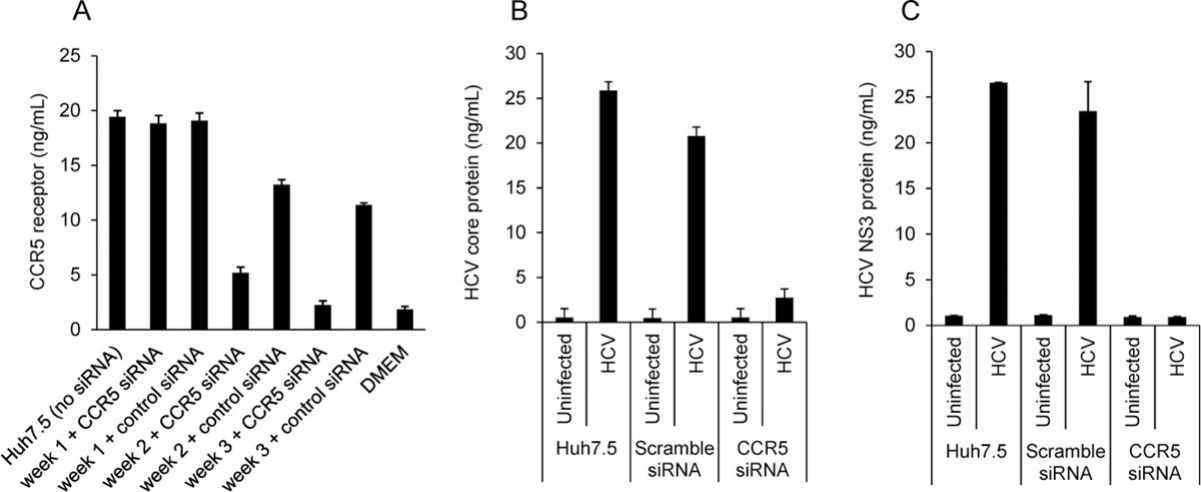
Effects of CCR5 knockdown. Huh7.5 cells were treated for 1–3 weeks with shRNA sequence corresponding to CCR5 or scramble to generate the Huh7.5_shCCR5_ and Huh7.5_shSCRAMBLE_ cell lines. **(A)** CCR5 receptor expression was evaluated using via a cell-based ELISA. Huh7.5_shCCR5_ or Huh7.5_shSCRAMBLE_ were plated in 24-well format and incubated with infectious virions harvested from the Huh7.5_JFH1_ cell line. HCV core protein **(B)** was quantified in cell culture supernatants, while HCV NS3 protein **(C)** was quantified in cell lysates.

To further evaluate the impact of CCR5 knockdown on HCV expression, the Huh7.5 cell line was incubated with shRNA sequence corresponding to CCR5 or scramble to generate the Huh7.5_shCCR5_ and Huh7.5_shSCRAMBLE_ cell lines. CCR5 receptor expression was evaluated using via a cell-based ELISA. As shown in Fig 4A, CCR5 expression was reduced ~75% by week 3 of shRNA treatment. Huh7.5_shCCR5_ or Huh7.5_shSCRAMBLE_ were plated in 24-well format and incubated with infectious virions harvested from the Huh7.5_JFH1_ cell line. A dramatic reduction in both HCV core and NS3 proteins were observed in the Huh7.5_shCCR5_ cell line compared to the Huh7.5_shSCRAMBLE_ or the parental Huh7.5 cell lines (Fig 4B and 4C).

**Fig 5:**
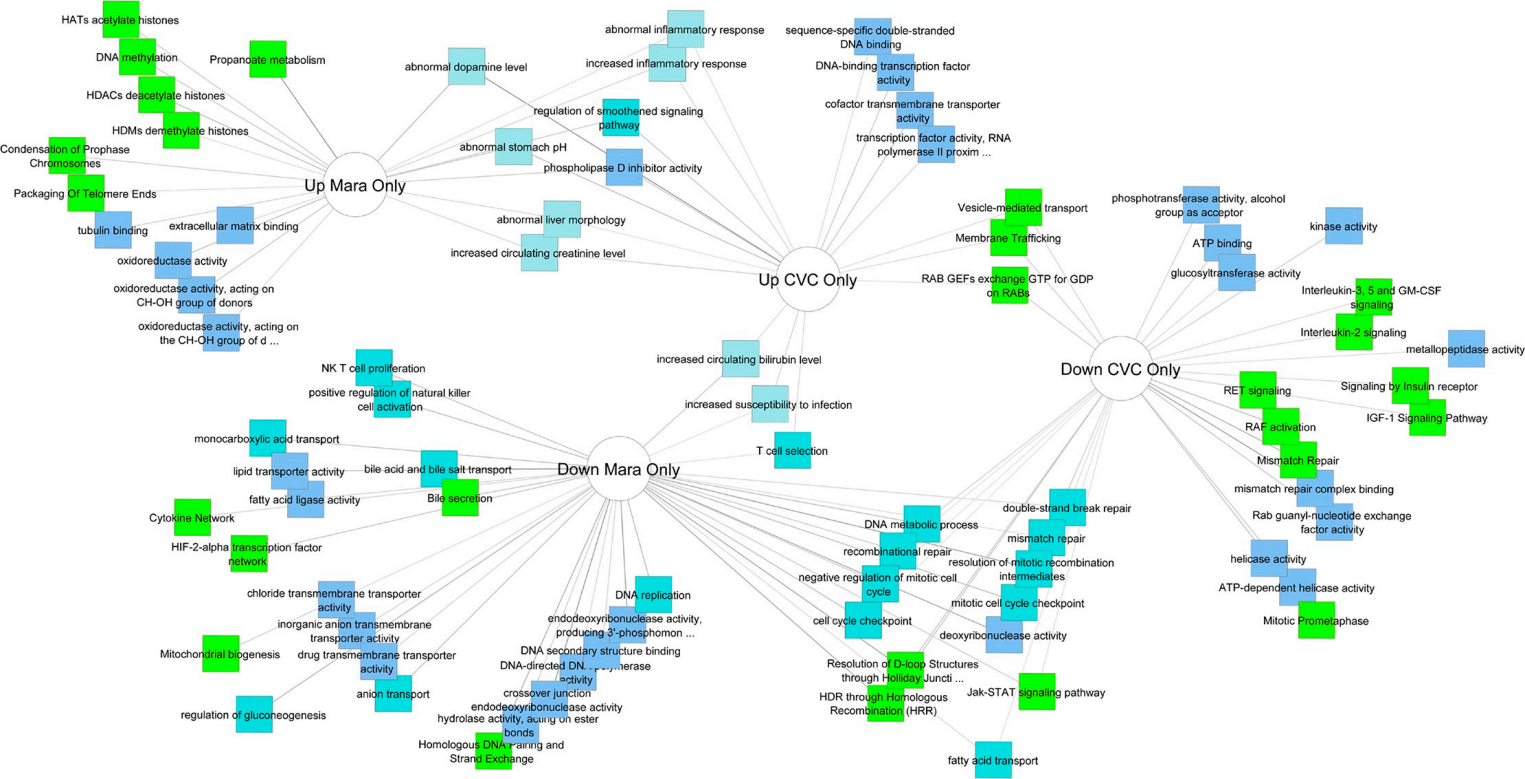
RNAseq analysis. Huh7.5_JFH1_ cells were exposed to sofosbuvir, cenicriviroc, maraviroc, or raltegravir for 24 hours. Cenicriviroc and maraviroc triplicate samples from day 1 post-drug incubation were compared to day 1 samples treated with sofosbuvir, with significance defined as p < 0.05 and fold-change > 1.5. Day 1 sofosbuvir-treated versus untreated samples were compared, and those genes from the cenicriviroc versus sofosbuvir and maraviroc versus sofosbuvir gene sets were removed to identify genes specific to the experimental treatments rather than the antiviral activity of sofosbuvir. Cenicriviroc and maraviroc-specific gene sets were submitted to Toppcluster (toppcluster.cchcm.org) and cytoscape for ontological analysis and network generation.

At day 1 post-drug incubation, genes from the cenicriviroc and maraviroc conditions were compared directly to the sofosbuvir condition to identify genes that are specific to the treatment effects of cenicriviroc and maraviroc and not the antiviral activity of sofosbuvir. As shown in Fig 5, several discrete functional gene clusters were identified that were either up- or down-regulated by both maraviroc and CVC. Common up-regulated genes included those related to inflammatory responses. Common down-regulated genes included those involved in fatty acid transport, Jak-STAT signaling, host cell-cycle regulation, and nucleic acid repair processes. A table of the up- and down-regulated genes that are treatment-specific for CVC and maraviroc relative to sofosbuvir is shown in S1A and S1B Table

## Discussion

Expression of HIV entry receptors has been explored in hepatocytes. Cao *et al*. found that the Huh7 and HepG2 hepatocyte-derived cell lines were CD4 negative [18], while others have reported that HepG2 cells were CD4 positive. [19, 20] Additional studies demonstrated that HepG2 cells also express the chemokine co-receptors CXCR4, CCR3, and CCR5.[20, 21] Vlahakis *et al*. reported that the Huh7, HepG2, and Hep3B cell lines, as well as primary hepatocytes, express CXCR4, although data on CD4 and other chemokine receptors were not provided.[22] Iser *et al*. were unable to detect CXCR4 or CCR5 on HepG2 cells via flow cytometry, although HIV infection of these cells could be blocked using CXCR4 or CCR5 antagonists.[23] Thus, low-level expression of CXCR4 and CCR5 receptors on hepatocytes seems likely, although flow cytometry may not be sufficiently sensitive for their detection. Additionally, several recent reports have convincingly demonstrated productive, low-level HIV infection of hepatocytes.[23–26]

In this report, we utilized two discrete lines of evidence to demonstrate that CCR5 plays a role in HCV replication in a cell-line based system. First, two different agents with clear CCR5 blockade capability provided a dose-dependent inhibition of HCV replication. One of the agents, cenicriviroc, also blocks CCR2 but the overall effect was similar to that seen for maraviroc which does not block CCR2, suggesting that CCR5 blockade is a key element of the observed outcomes. The level of decreased HCV replication was comparable to that observed for sofosbuvir, an inhibitor of HCV replication which antagonizes the RNA-dependent RNA polymerase. As expected, raltegravir–an integrase inhibitor widely used in patients with HIV – had no effect on HCV replication. Negative-strand HCV RNA was also decreased by the CCR5 inhibitors. Sandmann *et al*. suggested that pre-treatment of Huh-7.5 cells with maraviroc has a small but measurable inhibitory effect on HCV grown in culture. This effect was not directly related to virus entry or to downregulation of other known entry receptors.[27] Literature regarding the effect of maraviroc on HCV in clinical practice are limited. In a study of hepatic safety, Rockstroh *et al*. reported that HCV RNA decreased 3.22 IU/mL in HCV/HIV coinfected patients treated with maraviroc, although similar decreases were noted in the control arm.[28] In the setting of HIV suppression, HCV tends to first increase and then decrease to a lower setpoint[29]; thus, it is difficult to determine the effect of CCR5 blockade on HCV when both viruses have reciprocal effects on viral load. There are no data reporting effect of cenicriviroc on HCV replication as HCV has been an exclusion criteria in clinical trials of both HIV and NASH subjects.

The primary sources of chemokines in the liver are hepatocytes, Kupffer cells, stellate cells, sinusoidal endothelial cells, and biliary epithelial cells.[30] We observed only low or undetectable levels of RANTES protein in the Huh7.5_JFH1_ cell line, although others have detected it in Huh7.5 cells stably reconstituted with functional TLR3 signaling. [31] MIP1-alpha and MIP1-beta were expressed as noted in a previous study [32] [33], although no significant reduction in MIP expression was observed upon antiviral treatment of cells. Thus, altered expression of chemokine ligands is unlikely to play a major role in the CCR5 receptor-mediated blockade of HCV replication that was observed in our study.

Further evidence supporting an effect of CCR5 on HCV replication comes from inhibition with anti-CCR5 monoclonal antibodies and by CCR5 gene silencing studies. Fig 3 shows that two anti-CCR5 antibody preparations yielded the greatest decrease in core protein production. The use of gene silencing (siRNA) demonstrates clear differences compared to controls (Fig 4), in terms of both HCV core protein production and HCV NS3 protein production.

CCR5 blockade with cenicriviroc is clearly associated with a host of gene display variations which we previously reported.[14] However, the linkage of this effect to HCV replication has not yet been identified, and the mechanism(s) of HCV replication inhibition is unclear and is probably not directly related to a binding/entry process. CCR5 binds several cognate ligands including MIP-1 alpha and beta, as well as RANTES. These ligands are produced by hepatocytes in culture and are detectable in the supernatants of the cells used in these experiments (data not shown). It seems most like that the blockade of this cell-to-cell interaction leads to downstream effects on HCV replication. HCV replication and virion production is highly dependent upon adaptation of fatty acid synthesis pathways that facilitate the replication process. [34, 35] It is possible that down-regulation of genes involved with fatty acid synthesis which we observed inhibits HCV replication. In practical terms, HCV inhibition would be an added benefit for use of CCR5 blockade in HCV/HIV coinfected persons, as lower HCV RNA levels increase the chance of virologic cure. Further evaluation of CCR5 blockade in relation to HCV replication is warranted.

## Supporting information

S1 Table **Differentially regulated genes.** Up- and down-regulated genes treatment-specific for CVC (A) and maraviroc (B) relative to sofosbuvir.
(DOCX)

S1 Fig Chemokine measurement in cell culture. Expressed as fold change from day 1 no-drug control condition.
(TIF)

S2 Fig Supporting raw image for Fig 2A. Original annotated gel image for Fig 2A.
(PDF)

## References

[1] Michael NL, Nelson JAE, KewalRamani VN, Chang G, O'Brien SJ, Mascola JR, et al Exclusive and persistent use of the entry coreceptor CXCR4 by human immunodeficiency virus type 1 from a subject homozygous for CCR5 delta32. J Virol. 1998;72(7):6040–7. 9621067 10.1128/jvi.72.7.6040-6047.1998 PMC110409

[2] Marmor M, Sheppard HW, Donnell D, Bozeman S, Celum C, Buchbinder S, et al Homozygous and heterozygous CCR5-Delta32 genotypes are associated with resistance to HIV infection. Journal of acquired immune deficiency syndromes. 2001;27(5):472–81. 10.1097/00126334-200108150-00009 . 11511825

[3] Kostrikis LG, Huang Y, Moore JP, Wolinsky SM, Zhang L, Guo Y, et al A chemokine receptor CCR2 allele delays HIV-1 disease progression and is associated with a CCR5 promoter mutation. Nature medicine. 1998;4(3):350–3. 10.1038/nm0398-350 . 9500612

[4] Blackard JT, Kemmer N, Sherman K. Extrahepatic replication of HCV: insights into clinical manifestations and biological consequences. Hepatology. 2006;44(1):15–22. 10.1002/hep.21283 16799966

[5] Schwabe RF, Bataller R, Brenner DA. Human hepatic stellate cells express CCR5 and RANTES to induce proliferation and migration. Am J Physiol Gastrointest Liver Physiol. 2003;285(5):G949–58. Epub 2003/06/28. 10.1152/ajpgi.00215.2003 . 12829440

[6] Nomiyama H, Hieshima K, Nakayama T, Sakaguchi T, Fujisawa R, Tanase S, et al Human CC chemokine liver-expressed chemokine/CCL16 is a functional ligand for CCR1, CCR2 and CCR5, and constitutively expressed by hepatocytes. Int Immunol. 2001;13(8):1021–9. Epub 2001/07/27. 10.1093/intimm/13.8.1021 . 11470772

[7] Coenen M, Nattermann J. The role of CCR5 in HCV infection. Eur J Med Res. 2010;15(3):97–101. Epub 2010/05/11. 10.1186/2047-783X-15-3-97 20452893 PMC3352223

[8] Wasmuth HE, Werth A, Mueller T, Berg T, Dietrich CG, Geier A, et al CC chemokine receptor 5 delta32 polymorphism in two independent cohorts of hepatitis C virus infected patients without hemophilia. J Mol Med (Berl). 2004;82(1):64–9. Epub 2003/12/16. 10.1007/s00109-003-0505-0 . 14673528

[9] Ahlenstiel G, Woitas RP, Iwan A, Nattermann J, Feldmann G, Rockstroh JK, et al Effects of the CCR5-Delta32 mutation on hepatitis C virus-specific immune responses in patients with haemophilia. Immunol Invest. 2009;38(3–4):284–96. Epub 2009/10/09. . 19811439 10.1080/08820130902832035

[10] Wald O, Pappo O, Ari ZB, Azzaria E, Wiess ID, Gafnovitch I, et al The CCR5 Delta 32 allele is associated with reduced liver inflammation in hepatitis C virus infection. Eur J Immunogenet. 2004;31(6):249–52. 10.1111/j.1365-2370.2004.00482.x WOS:000225151500002. 15548261

[11] Rockstroh JK, Plonski F, Bansal M, Fatkenheuer G, Small CB, Asmuth DM, et al Hepatic safety of maraviroc in patients with HIV-1 and hepatitis C and/or B virus: 144-week results from a randomized, placebo-controlled trial. Antiviral therapy. 2017;22(3):263–9. Epub 2016/12/08. 10.3851/IMP3116 . 27924779

[12] Friedman SL, Ratziu V, Harrison SA, Abdelmalek MF, Aithal GP, Caballeria J, et al A randomized, placebo-controlled trial of cenicriviroc for treatment of nonalcoholic steatohepatitis with fibrosis. Hepatology. 2017 10.1002/hep.29477 . 28833331 PMC5947654

[13] Thompson M, Saag M, DeJesus E, Gathe J, Lalezari J, Landay AL, et al A 48-week randomized phase 2b study evaluating cenicriviroc versus efavirenz in treatment-naive HIV-infected adults with C-C chemokine receptor type 5-tropic virus. Aids. 2016;30(6):869–78. 10.1097/QAD.0000000000000988 26636929 PMC4794136

[14] Sherman KE, Abdel-Hameed E, Rouster SD, Shata MTM, Blackard JT, Safaie P, et al Improvement in Hepatic Fibrosis Biomarkers Associated with Chemokine Receptor Inactivation through Mutation or Therapeutic Blockade. Clinical infectious diseases: an official publication of the Infectious Diseases Society of America. 2018 Epub 2018/09/22. 10.1093/cid/ciy807 . 30239650 PMC6784280

[15] Cai Z, Zhang C, Chang KS, Jiang J, Ahn BC, Wakita T, et al Robust production of infectious hepatitis C virus (HCV) from stably HCV cDNA-transfected human hepatoma cells. Journal of Virology. 2005;79(22):13963–73. 10.1128/JVI.79.22.13963-13973.2005 . 16254332 PMC1280219

[16] Blackard JT, Kong L, Huber AK, Tomer Y. Hepatitis C virus infection of a thyroid cell line: implications for pathogenesis of hepatitis C virus and thyroiditis. Thyroid. 2013;23(7):863–70. 10.1089/thy.2012.0507 23259732 PMC3704108

[17] Hiasa Y, Blackard JT, Lin W, Kamegaya Y, Horiike N, Onji M, et al Cell-based models of sustained, interferon-sensitive hepatitis C virus genotype 1 replication. Journal of Virologic Methods. 2006;132:195–203. 10.1016/j.jviromet.2005.10.014 PMC2865175 16313977

[18] Cao YZ, Friedman-Kein AE, Huang YX, Li XL, Mirabile M, Moudgil T, et al CD4-independent, productive human immunodeficiency virus type 1 infection of hepatoma cell lines in vitro. Journal of Virology. 1990;64(6):2553–9. 2159530 10.1128/jvi.64.6.2553-2559.1990 PMC249431

[19] Banerjee R, Sperber K, Pizzella T, Mayer L. Inhibition of HIV-1 productive infection in hepatoblastoma HepG2 cells by recombinant tumor necrosis factor-a. Aids. 1992;6(10):1127–31. 10.1097/00002030-199210000-00010 1466843

[20] Munshi N, Balasubramanian A, Koziel M, Ganju R, Groopman J. Hepatitis C and human immunodeficiency virus envelope proteins cooperatively induce hepatocytic apoptosis via an innocent bystander mechanism. Journal of Infectious Diseases. 2003;188:1192–204. 10.1086/378643 14551890

[21] Balasubramanian A, Ganju R, Groopman J. HCV and HIV envelope proteins collaboratively mediate IL-8 secretion through activation of p38 MAP kinase and SHP2 in hepatocytes. Journal of Biological Chemistry. 2003;278(37):35755–66. 10.1074/jbc.M302889200 12824191

[22] Vlahakis S, Villasis-Keever A, Gomez T, Bren G, Paya C. Human immunodeficiency virus-induced apoptosis of human hepatocytes via CXCR4. Journal of Infectious Diseases. 2003;188:1455–60. 10.1086/379738 14624370

[23] Iser DM, Warner N, Revill PA, Solomon A, Wightman F, Saleh S, et al Coinfection of hepatic cell lines with human immunodeficiency virus and hepatitis B virus leads to an increase in intracellular hepatitis B surface antigen. Journal of Virology. 2010;84(12):5860–7. 10.1128/JVI.02594-09 20357083 PMC2876638

[24] Xiao P, Usami O, Suzuki Y, Ling H, Shimizu N, Hoshino H, et al Characterization of a CD4-independent clinical HIV-1 that can efficiently infect human hepatocytes through chemokine (C-X-C motif) receptor 4. AIDS. 2008;22(14):1749–57. 10.1097/QAD.0b013e328308937c 18753859

[25] Fromentin R, MR T, Tremblay M. Human hepatoma cells transmit surface bound HIV-1 to CD4+ T cells through an ICAM-1/LFA-1-dependent mechanism. Virology. 2010;398:168–75. 10.1016/j.virol.2009.12.008 20034651

[26] Kong L, Cardona Maya W, Moreno-Fernandez ME, Ma G SM, Sherman KE, Chougnet C, et al Low-level HIV infection of hepatocytes. Virology Journal. 2012;9(1):157. 22877244 10.1186/1743-422X-9-157 PMC3607931

[27] Sandmann L, Wilson M, Back D, Wedemeyer H, Manns MP, Steinmann E, et al Anti-retroviral drugs do not facilitate hepatitis C virus (HCV) infection in vitro. Antiviral research. 2012;96(1):51–8. Epub 2012/07/31. 10.1016/j.antiviral.2012.07.005 . 22842003

[28] Rockstroh JK, Soriano V, Plonski F, Bansal M, Fatkenheuer G, Small CB, et al Hepatic safety in subjects with HIV-1 and hepatitis C and/or B virus: a randomized, double-blind study of maraviroc versus placebo in combination with antiretroviral agents. HIV Clin Trials. 2015;16(2):72–80. Epub 2015/04/30. 10.1179/1528433614Z.0000000011 . 25923596

[29] Sherman KE, Guedj J, Shata MT, Blackard JT, Rouster SD, Castro M, et al Modulation of HCV replication after combination antiretroviral therapy in HCV/HIV co-infected patients. Science translational medicine. 2014;6(246):246ra98. 10.1126/scitranslmed.3008195 25101888 PMC4326686

[30] Saiman Y, Friedman SL. The role of chemokines in acute liver injury. Front Physiol. 2012;3:213 Epub 2012/06/23. 10.3389/fphys.2012.00213 22723782 PMC3379724

[31] Li K, Li NL, Wei D, Pfeffer SR, Fan M, Pfeffer LM. Activation of chemokine and inflammatory cytokine response in hepatitis C virus-infected hepatocytes depends on Toll-like receptor 3 sensing of hepatitis C virus double-stranded RNA intermediates. Hepatology. 2012;55(3):666–75. Epub 2011/10/28. 10.1002/hep.24763 22030901 PMC3272326

[32] Nishitsuji H, Funami K, Shimizu Y, Ujino S, Sugiyama K, Seya T, et al Hepatitis C virus infection induces inflammatory cytokines and chemokines mediated by the cross talk between hepatocytes and stellate cells. Journal of virology. 2013;87(14):8169–78. Epub 2013/05/17. 10.1128/JVI.00974-13 23678168 PMC3700210

[33] Zhang T, Guo CJ, Li Y, Douglas SD, Qi XX, Song L, et al Interleukin-1beta induces macrophage inflammatory protein-1beta expression in human hepatocytes. Cell Immunol. 2003;226(1):45–53. Epub 2004/01/30. 10.1016/j.cellimm.2003.10.005 14746807 PMC4016814

[34] Hofmann S, Krajewski M, Scherer C, Scholz V, Mordhorst V, Truschow P, et al Complex lipid metabolic remodeling is required for efficient hepatitis C virus replication. Biochim Biophys Acta Mol Cell Biol Lipids. 2018;1863(9):1041–56. Epub 2018/06/10. 10.1016/j.bbalip.2018.06.002 . 29885363

[35] Vieyres G, Pietschmann T. HCV Pit Stop at the Lipid Droplet: Refuel Lipids and Put on a Lipoprotein Coat before Exit. Cells. 2019;8(3). Epub 2019/03/16. 10.3390/cells8030233 30871009 PMC6468556

