# Supplementary material for: CCR5 receptor antagonism inhibits hepatitis C virus (HCV) replication in vitro
Source: PLoS One. 2019 Oct 29;14(10):e0224523. doi: 10.1371/journal.pone.0224523 (PMC6818973; doi:10.1371/journal.pone.0224523)
Supplement: S1 Table — Differentially regulated genes. Up- and down-regulated genes treatment-specific for CVC (A) and maraviroc (B) relative to sofosbuvir. (DOCX) [file pone.0224523.s001.docx]

**S1 Table A**: **Differentially regulated genes.** Up- and down-regulated CVC-specific genes relative to sofosbuvir. Fold change is >= 1.25.

| **Gene** | **Fold Change ([Day1 CVC] vs [Day1 Sof])** | **Log Fold Change ([Day1 CVC] vs [Day1 Sof])** | **Fold Change absolute ([Day1 CVC] vs [Day1 Sof])** | **Regulation ([Day1 CVC] vs [Day1 Sof])** |
| --- | --- | --- | --- | --- |
| CCDC39 | -2.4566028 | -1.2966646 | 2.4566028 | down |
| DNASE1L1 | -2.3552651 | -1.2358894 | 2.3552651 | down |
| TREX1 | -2.2560236 | -1.1737822 | 2.2560236 | down |
| RFESD | -2.0316224 | -1.0226324 | 2.0316224 | down |
| CFAP69 | -1.9757048 | -0.9823674 | 1.9757048 | down |
| MTM1 | -1.8829997 | -0.91303277 | 1.8829997 | down |
| MCTP1 | -1.8654591 | -0.8995307 | 1.8654591 | down |
| KSR1 | -1.8587786 | -0.89435494 | 1.8587786 | down |
| SEMA3F | -1.8166436 | -0.86127543 | 1.8166436 | down |
| ANKRD36B | -1.777272 | -0.82966447 | 1.777272 | down |
| TAS2R43 | -1.7375258 | -0.79703444 | 1.7375258 | down |
| CCDC82 | -1.694586 | -0.7609329 | 1.694586 | down |
| TMEM150C | -1.6805506 | -0.7489339 | 1.6805506 | down |
| DEPDC7 | -1.6754072 | -0.7445117 | 1.6754072 | down |
| COLQ | -1.6599038 | -0.7310996 | 1.6599038 | down |
| SLC12A6 | -1.6413841 | -0.71491295 | 1.6413841 | down |
| NIPSNAP3B | -1.6294719 | -0.7044045 | 1.6294719 | down |
| RBM44 | -1.6261501 | -0.7014604 | 1.6261501 | down |
| SLC27A5 | -1.6213293 | -0.6971772 | 1.6213293 | down |
| SCNN1A | -1.6059363 | -0.6834147 | 1.6059363 | down |
| DYRK1B | -1.6003083 | -0.6783499 | 1.6003083 | down |
| SPATA7 | -1.5966302 | -0.6750302 | 1.5966302 | down |
| DDX17 | -1.5872507 | -0.66653 | 1.5872507 | down |
| ANO5 | -1.5759273 | -0.65620095 | 1.5759273 | down |
| ADAM28 | -1.5579888 | -0.63968486 | 1.5579888 | down |
| ERCC3 | -1.5344121 | -0.6176861 | 1.5344121 | down |
| CAPS2 | -1.5213696 | -0.60537064 | 1.5213696 | down |
| TTC3 | -1.5195516 | -0.6036457 | 1.5195516 | down |
| E2F7 | -1.5107499 | -0.59526485 | 1.5107499 | down |
| UBR7 | -1.4887162 | -0.57406884 | 1.4887162 | down |
| FAN1 | -1.4862424 | -0.57166946 | 1.4862424 | down |
| TWSG1 | -1.4742227 | -0.5599544 | 1.4742227 | down |
| L3MBTL3 | -1.4727938 | -0.5585554 | 1.4727938 | down |
| ZNF337 | -1.4504985 | -0.53654873 | 1.4504985 | down |
| ZNF525 | -1.4475776 | -0.53364074 | 1.4475776 | down |
| CCDC180 | -1.4421083 | -0.5281795 | 1.4421083 | down |
| ZER1 | -1.4413743 | -0.527445 | 1.4413743 | down |
| CDCA2 | -1.4410707 | -0.5271411 | 1.4410707 | down |
| CDKN2AIP | -1.4369388 | -0.52299863 | 1.4369388 | down |
| C5orf34 | -1.4334121 | -0.5194534 | 1.4334121 | down |
| WDR89 | -1.4297298 | -0.5157426 | 1.4297298 | down |
| MAP3K13 | -1.4177616 | -0.5036149 | 1.4177616 | down |
| PRR4 | -1.4145113 | -0.5003037 | 1.4145113 | down |
| PPP2R5A | -1.4137528 | -0.49952984 | 1.4137528 | down |
| MREG | -1.4118674 | -0.4976046 | 1.4118674 | down |
| C3orf35 | -1.40804 | -0.49368832 | 1.40804 | down |
| SLC2A13 | -1.4022162 | -0.48770875 | 1.4022162 | down |
| ZNF699 | -1.3945444 | -0.47979382 | 1.3945444 | down |
| TSTD3 | -1.3944799 | -0.47972712 | 1.3944799 | down |
| C1QTNF3 | -1.3922131 | -0.4773801 | 1.3922131 | down |
| COPS4 | -1.3910156 | -0.4761386 | 1.3910156 | down |
| DPY19L4 | -1.389517 | -0.47458348 | 1.389517 | down |
| RIC1 | -1.382412 | -0.46718764 | 1.382412 | down |
| ZNF680 | -1.3807523 | -0.46545458 | 1.3807523 | down |
| RTTN | -1.3801317 | -0.464806 | 1.3801317 | down |
| TMEM251 | -1.3778971 | -0.46246815 | 1.3778971 | down |
| DDHD2 | -1.3778955 | -0.4624664 | 1.3778955 | down |
| ZKSCAN3 | -1.3736898 | -0.4580562 | 1.3736898 | down |
| SLC9A6 | -1.3731616 | -0.4575014 | 1.3731616 | down |
| GAPVD1 | -1.3725262 | -0.45683366 | 1.3725262 | down |
| FAM168A | -1.3705118 | -0.45471472 | 1.3705118 | down |
| DDX47 | -1.3686293 | -0.45273176 | 1.3686293 | down |
| AGTRAP | -1.361756 | -0.4454682 | 1.361756 | down |
| GNG10 | -1.3596718 | -0.44325846 | 1.3596718 | down |
| MYO6 | -1.3580976 | -0.44158712 | 1.3580976 | down |
| TBC1D2B | -1.3557252 | -0.4390648 | 1.3557252 | down |
| C16orf87 | -1.3556648 | -0.4390006 | 1.3556648 | down |
| MORN2 | -1.352591 | -0.43572575 | 1.352591 | down |
| CEP192 | -1.3520107 | -0.4351066 | 1.3520107 | down |
| NRDE2 | -1.350968 | -0.4339935 | 1.350968 | down |
| IL13RA1 | -1.3504207 | -0.43340892 | 1.3504207 | down |
| AGTPBP1 | -1.3461206 | -0.42880765 | 1.3461206 | down |
| STK36 | -1.3457519 | -0.42841244 | 1.3457519 | down |
| ZWINT | -1.3431488 | -0.42561913 | 1.3431488 | down |
| HEATR5A | -1.3406025 | -0.4228816 | 1.3406025 | down |
| EID1 | -1.3390442 | -0.4212036 | 1.3390442 | down |
| TMEM161B | -1.3378369 | -0.41990218 | 1.3378369 | down |
| CCDC7 | -1.3376144 | -0.41966236 | 1.3376144 | down |
| TMEM17 | -1.3367468 | -0.4187262 | 1.3367468 | down |
| RIOK2 | -1.3341093 | -0.41587687 | 1.3341093 | down |
| TAF2 | -1.3338529 | -0.4155995 | 1.3338529 | down |
| USP32 | -1.3329792 | -0.41465425 | 1.3329792 | down |
| MLH1 | -1.3324316 | -0.4140614 | 1.3324316 | down |
| NMRK1 | -1.3308452 | -0.4123428 | 1.3308452 | down |
| C12orf29 | -1.3297758 | -0.41118303 | 1.3297758 | down |
| TDRKH | -1.3295583 | -0.41094697 | 1.3295583 | down |
| IREB2 | -1.3291193 | -0.41047063 | 1.3291193 | down |
| SPTBN5 | -1.3287349 | -0.41005322 | 1.3287349 | down |
| LMAN1 | -1.328574 | -0.40987858 | 1.328574 | down |
| MED28 | -1.3266934 | -0.407835 | 1.3266934 | down |
| ZNF326 | -1.3258908 | -0.40696192 | 1.3258908 | down |
| ISCA2 | -1.3250881 | -0.40608835 | 1.3250881 | down |
| PPP4R1 | -1.3225799 | -0.40335482 | 1.3225799 | down |
| EYA3 | -1.3183777 | -0.39876372 | 1.3183777 | down |
| RBKS | -1.3169779 | -0.3972311 | 1.3169779 | down |
| ZNF512 | -1.3156351 | -0.39575943 | 1.3156351 | down |
| GEN1 | -1.3130184 | -0.39288718 | 1.3130184 | down |
| DENND1B | -1.3129431 | -0.39280444 | 1.3129431 | down |
| SIL1 | -1.3104298 | -0.39004007 | 1.3104298 | down |
| SMDT1 | -1.3103695 | -0.38997364 | 1.3103695 | down |
| DHX8 | -1.3090761 | -0.388549 | 1.3090761 | down |
| TRMT2B | -1.3081735 | -0.38755387 | 1.3081735 | down |
| AP5S1 | -1.3058348 | -0.38497233 | 1.3058348 | down |
| SMC4 | -1.3000374 | -0.37855306 | 1.3000374 | down |
| HOXA10 | -1.2994554 | -0.37790713 | 1.2994554 | down |
| ANKRD36C | -1.2993497 | -0.37778968 | 1.2993497 | down |
| RNPEP | -1.2989897 | -0.3773899 | 1.2989897 | down |
| RPAP1 | -1.2976137 | -0.37586102 | 1.2976137 | down |
| TM9SF2 | -1.2972372 | -0.3754422 | 1.2972372 | down |
| DCBLD1 | -1.2966409 | -0.37477893 | 1.2966409 | down |
| ADI1 | -1.2965844 | -0.37471613 | 1.2965844 | down |
| EIF2AK4 | -1.2962589 | -0.37435398 | 1.2962589 | down |
| PRKAB2 | -1.2949206 | -0.37286362 | 1.2949206 | down |
| TSG101 | -1.2941382 | -0.37199163 | 1.2941382 | down |
| FASTKD3 | -1.2938484 | -0.37166852 | 1.2938484 | down |
| ZNF695 | -1.292732 | -0.37042326 | 1.292732 | down |
| SNX29 | -1.2918236 | -0.36940908 | 1.2918236 | down |
| EFR3A | -1.2915101 | -0.36905894 | 1.2915101 | down |
| ERCC6L | -1.2913761 | -0.36890924 | 1.2913761 | down |
| TRAPPC4 | -1.2908746 | -0.3683488 | 1.2908746 | down |
| STAMBPL1 | -1.2885048 | -0.36569795 | 1.2885048 | down |
| CIPC | -1.2875495 | -0.36462784 | 1.2875495 | down |
| LYSMD1 | -1.2862732 | -0.3631971 | 1.2862732 | down |
| ITFG1 | -1.2860781 | -0.36297828 | 1.2860781 | down |
| RAF1 | -1.2830176 | -0.35954094 | 1.2830176 | down |
| U2SURP | -1.2825067 | -0.35896635 | 1.2825067 | down |
| ZNF43 | -1.2823064 | -0.35874104 | 1.2823064 | down |
| HOXC4 | -1.2811316 | -0.35741872 | 1.2811316 | down |
| ABCC4 | -1.280055 | -0.35620585 | 1.280055 | down |
| ZNF736 | -1.2795484 | -0.35563472 | 1.2795484 | down |
| ACTR2 | -1.2785696 | -0.35453063 | 1.2785696 | down |
| PIK3R1 | -1.2782794 | -0.35420322 | 1.2782794 | down |
| PIAS2 | -1.2777926 | -0.3536536 | 1.2777926 | down |
| GOLGA8B | -1.2775711 | -0.35340357 | 1.2775711 | down |
| EME1 | -1.2770727 | -0.3528406 | 1.2770727 | down |
| KIAA2026 | -1.2747372 | -0.35019988 | 1.2747372 | down |
| UGCG | -1.2742064 | -0.34959903 | 1.2742064 | down |
| SIKE1 | -1.2736158 | -0.34893018 | 1.2736158 | down |
| ZBTB26 | -1.273261 | -0.34852818 | 1.273261 | down |
| GLT8D1 | -1.2723787 | -0.34752813 | 1.2723787 | down |
| CAMLG | -1.2721585 | -0.34727842 | 1.2721585 | down |
| LSG1 | -1.2699275 | -0.3447461 | 1.2699275 | down |
| F11R | -1.2698389 | -0.3446455 | 1.2698389 | down |
| DZIP3 | -1.2681001 | -0.3426687 | 1.2681001 | down |
| TXN2 | -1.2658044 | -0.3400545 | 1.2658044 | down |
| ZRANB1 | -1.265489 | -0.3396949 | 1.265489 | down |
| ZC3HAV1 | -1.2649059 | -0.3390301 | 1.2649059 | down |
| ZDHHC23 | -1.2615354 | -0.3351807 | 1.2615354 | down |
| PARM1 | -1.2614478 | -0.33508047 | 1.2614478 | down |
| PCNA | -1.2608318 | -0.33437586 | 1.2608318 | down |
| HMGXB4 | -1.2604307 | -0.33391675 | 1.2604307 | down |
| CCNDBP1 | -1.2601249 | -0.3335668 | 1.2601249 | down |
| RCHY1 | -1.2599288 | -0.33334225 | 1.2599288 | down |
| EXO1 | -1.2589158 | -0.33218178 | 1.2589158 | down |
| SHOC2 | -1.2582946 | -0.33146968 | 1.2582946 | down |
| NRCAM | -1.2576361 | -0.33071455 | 1.2576361 | down |
| ZYG11A | -1.2561104 | -0.32896328 | 1.2561104 | down |
| RIOK1 | -1.2560949 | -0.3289455 | 1.2560949 | down |
| AKAP1 | -1.2555209 | -0.32828602 | 1.2555209 | down |
| PLS1 | -1.2550843 | -0.32778424 | 1.2550843 | down |
| HADHB | -1.2540329 | -0.32657513 | 1.2540329 | down |
| PPWD1 | -1.2539973 | -0.32653427 | 1.2539973 | down |
| FMO5 | -1.2530276 | -0.32541808 | 1.2530276 | down |
| CMPK1 | -1.2512026 | -0.32331544 | 1.2512026 | down |
| PSMD7 | -1.2508649 | -0.3229259 | 1.2508649 | down |
| FAH | 1.2528534 | 0.32521757 | 1.2528534 | up |
| TRAPPC2L | 1.2577916 | 0.3308929 | 1.2577916 | up |
| AFF2 | 1.2623945 | 0.33616292 | 1.2623945 | up |
| MCM5 | 1.26723 | 0.34167838 | 1.26723 | up |
| ACY1 | 1.2686584 | 0.34330368 | 1.2686584 | up |
| ZNF283 | 1.2707521 | 0.34568256 | 1.2707521 | up |
| OCA2 | 1.2745531 | 0.34999144 | 1.2745531 | up |
| AHDC1 | 1.2758642 | 0.35147485 | 1.2758642 | up |
| C17orf80 | 1.275887 | 0.3515006 | 1.275887 | up |
| CTDP1 | 1.2946986 | 0.3726163 | 1.2946986 | up |
| DTD1 | 1.3016427 | 0.38033342 | 1.3016427 | up |
| MCOLN1 | 1.3018599 | 0.38057417 | 1.3018599 | up |
| MRPL20 | 1.3041518 | 0.3831118 | 1.3041518 | up |
| DHRS13 | 1.3051337 | 0.38419756 | 1.3051337 | up |
| C22orf46 | 1.3063143 | 0.38550213 | 1.3063143 | up |
| SLC25A26 | 1.3098117 | 0.38935938 | 1.3098117 | up |
| ZNF629 | 1.3100396 | 0.38961053 | 1.3100396 | up |
| GPATCH1 | 1.310107 | 0.38968468 | 1.310107 | up |
| PDE1A | 1.3158826 | 0.3960307 | 1.3158826 | up |
| KTI12 | 1.3182857 | 0.3986631 | 1.3182857 | up |
| SHH | 1.3204905 | 0.4010739 | 1.3204905 | up |
| KAZALD1 | 1.3344762 | 0.41627362 | 1.3344762 | up |
| ZNF768 | 1.3351693 | 0.4170227 | 1.3351693 | up |
| TAF6L | 1.3416132 | 0.42396873 | 1.3416132 | up |
| ZBTB21 | 1.3418325 | 0.4242046 | 1.3418325 | up |
| RFXAP | 1.3450527 | 0.42766267 | 1.3450527 | up |
| C2orf15 | 1.347277 | 0.43004656 | 1.347277 | up |
| DNAAF5 | 1.3487471 | 0.43161994 | 1.3487471 | up |
| ZNF516 | 1.3501899 | 0.4331624 | 1.3501899 | up |
| ZNF696 | 1.353159 | 0.43633133 | 1.353159 | up |
| PODN | 1.3614293 | 0.4451221 | 1.3614293 | up |
| SPSB2 | 1.3622438 | 0.4459849 | 1.3622438 | up |
| TMPRSS5 | 1.3640445 | 0.4478908 | 1.3640445 | up |
| DBP | 1.3730903 | 0.4574265 | 1.3730903 | up |
| MMAB | 1.3750983 | 0.45953482 | 1.3750983 | up |
| SH3KBP1 | 1.3854322 | 0.4703361 | 1.3854322 | up |
| GLIS2 | 1.385676 | 0.47059003 | 1.385676 | up |
| PLAU | 1.3877702 | 0.47276872 | 1.3877702 | up |
| SPINK5 | 1.3913717 | 0.47650796 | 1.3913717 | up |
| URB1 | 1.3989421 | 0.48433623 | 1.3989421 | up |
| RAB17 | 1.4020652 | 0.48755345 | 1.4020652 | up |
| UBE2L6 | 1.4098874 | 0.49557996 | 1.4098874 | up |
| PRKCH | 1.4121352 | 0.4978783 | 1.4121352 | up |
| UNC93B1 | 1.4128253 | 0.49858308 | 1.4128253 | up |
| C4orf3 | 1.4175678 | 0.5034178 | 1.4175678 | up |
| TMSB4X | 1.4216456 | 0.5075619 | 1.4216456 | up |
| POU6F2 | 1.4389145 | 0.52498096 | 1.4389145 | up |
| NDUFA7 | 1.4414983 | 0.5275691 | 1.4414983 | up |
| CD3D | 1.4606166 | 0.5465775 | 1.4606166 | up |
| NEDD8 | 1.4647946 | 0.5506984 | 1.4647946 | up |
| FLVCR2 | 1.4673928 | 0.5532551 | 1.4673928 | up |
| RIN2 | 1.471078 | 0.55687374 | 1.471078 | up |
| NXPH4 | 1.4816545 | 0.5672091 | 1.4816545 | up |
| HCFC1R1 | 1.4895555 | 0.5748819 | 1.4895555 | up |
| ZNF394 | 1.5001942 | 0.5851493 | 1.5001942 | up |
| RPL36AL | 1.5054759 | 0.5902196 | 1.5054759 | up |
| RAB37 | 1.5313917 | 0.6148434 | 1.5313917 | up |
| IFT140 | 1.5411319 | 0.6239903 | 1.5411319 | up |
| C1orf123 | 1.5446315 | 0.6272627 | 1.5446315 | up |
| COX6C | 1.5486144 | 0.630978 | 1.5486144 | up |
| TMEM256 | 1.5513176 | 0.633494 | 1.5513176 | up |
| TEF | 1.551939 | 0.6340719 | 1.551939 | up |
| TFIP11 | 1.5640954 | 0.64532846 | 1.5640954 | up |
| C19orf70 | 1.574208 | 0.6546262 | 1.574208 | up |
| PLCD4 | 1.583222 | 0.6628636 | 1.583222 | up |
| TNNC1 | 1.6095395 | 0.686648 | 1.6095395 | up |
| CCDC102A | 1.6660942 | 0.7364699 | 1.6660942 | up |
| AKNA | 1.6663356 | 0.73667896 | 1.6663356 | up |
| HIST2H2AA4 | 1.7124104 | 0.7760285 | 1.7124104 | up |
| SNCA | 1.8029346 | 0.8503471 | 1.8029346 | up |
| GPR157 | 1.8177114 | 0.86212313 | 1.8177114 | up |
| EBF4 | 1.8716159 | 0.90428436 | 1.8716159 | up |
| RAB43 | 1.8725827 | 0.9050294 | 1.8725827 | up |
| ESPN | 1.8847897 | 0.91440356 | 1.8847897 | up |
| UNC13D | 3.1010623 | 1.6327626 | 3.1010623 | up |

**S1 Table B**: **Differentially regulated genes.** Up- and down-regulated Maraviroc-specific genes relative to sofosbuvir. Fold change is >= 1.25.

| **Gene** | **Fold Change ([Day1 Mara] vs [Day1 Sof])** | | **Log Fold Change ([Day1 Mara] vs [Day1 Sof])** | | **Fold Change absolute ([Day1 Mara] vs [Day1 Sof])** | | **Regulation ([Day1 Mara] vs [Day1 Sof])** | | |
| --- | --- | --- | --- | --- | --- | --- | --- | --- | --- |
| CDKL1 | | -4.6978707 | | -2.232007 | | 4.6978707 | | down |  |
| DUX4 | | -2.6801684 | | -1.4223236 | | 2.6801684 | | down |  |
| SPDYA | | -2.4386861 | | -1.2861041 | | 2.4386861 | | down |  |
| ELOVL6 | | -2.1174943 | | -1.0823581 | | 2.1174943 | | down |  |
| TCTEX1D1 | | -1.999369 | | -0.99954474 | | 1.999369 | | down |  |
| FOXO4 | | -1.9920064 | | -0.9942223 | | 1.9920064 | | down |  |
| TREX1 | | -1.956767 | | -0.96847194 | | 1.956767 | | down |  |
| KHDRBS3 | | -1.9434444 | | -0.9586158 | | 1.9434444 | | down |  |
| KMO | | -1.8999674 | | -0.92597467 | | 1.8999674 | | down |  |
| KRTAP19-5 | | -1.8349105 | | -0.8757097 | | 1.8349105 | | down |  |
| ABCB4 | | -1.8255186 | | -0.8683064 | | 1.8255186 | | down |  |
| ANKRD36B | | -1.8044627 | | -0.8515693 | | 1.8044627 | | down |  |
| DYNC1I1 | | -1.7993861 | | -0.84750485 | | 1.7993861 | | down |  |
| MOSPD2 | | -1.7647588 | | -0.819471 | | 1.7647588 | | down |  |
| XRCC3 | | -1.7589742 | | -0.81473434 | | 1.7589742 | | down |  |
| TAS2R43 | | -1.7586981 | | -0.81450784 | | 1.7586981 | | down |  |
| SLC27A5 | | -1.745172 | | -0.8033693 | | 1.745172 | | down |  |
| SCGB3A2 | | -1.6887052 | | -0.75591755 | | 1.6887052 | | down |  |
| PRPSAP2 | | -1.6664164 | | -0.736749 | | 1.6664164 | | down |  |
| ACSM1 | | -1.6296238 | | -0.7045389 | | 1.6296238 | | down |  |
| ZNF532 | | -1.620726 | | -0.6966402 | | 1.620726 | | down |  |
| TNFRSF11A | | -1.6088817 | | -0.6860582 | | 1.6088817 | | down |  |
| SLC39A8 | | -1.5906198 | | -0.66958904 | | 1.5906198 | | down |  |
| C3orf35 | | -1.5789863 | | -0.65899867 | | 1.5789863 | | down |  |
| TSGA10 | | -1.576563 | | -0.6567828 | | 1.576563 | | down |  |
| TTC26 | | -1.5701641 | | -0.6509154 | | 1.5701641 | | down |  |
| AGO3 | | -1.5552382 | | -0.6371356 | | 1.5552382 | | down |  |
| FAM168A | | -1.552526 | | -0.63461745 | | 1.552526 | | down |  |
| IFNAR2 | | -1.5522058 | | -0.6343199 | | 1.5522058 | | down |  |
| PDE8A | | -1.5503738 | | -0.63261604 | | 1.5503738 | | down |  |
| GEN1 | | -1.5488526 | | -0.6311998 | | 1.5488526 | | down |  |
| SLC12A6 | | -1.5192477 | | -0.6033571 | | 1.5192477 | | down |  |
| RAD51C | | -1.5111552 | | -0.5956519 | | 1.5111552 | | down |  |
| JMY | | -1.5076306 | | -0.59228295 | | 1.5076306 | | down |  |
| CLHC1 | | -1.4917041 | | -0.5769614 | | 1.4917041 | | down |  |
| MDGA2 | | -1.4904406 | | -0.5757389 | | 1.4904406 | | down |  |
| FIGN | | -1.4808115 | | -0.56638795 | | 1.4808115 | | down |  |
| MON2 | | -1.4791607 | | -0.5647788 | | 1.4791607 | | down |  |
| ANO5 | | -1.4711379 | | -0.55693245 | | 1.4711379 | | down |  |
| HEATR3 | | -1.4709117 | | -0.5567107 | | 1.4709117 | | down |  |
| EDEM3 | | -1.4690832 | | -0.5549161 | | 1.4690832 | | down |  |
| SLCO2B1 | | -1.4673458 | | -0.55320895 | | 1.4673458 | | down |  |
| C2orf88 | | -1.4598176 | | -0.5457881 | | 1.4598176 | | down |  |
| TIAL1 | | -1.4551795 | | -0.5411971 | | 1.4551795 | | down |  |
| GAPVD1 | | -1.447223 | | -0.5332872 | | 1.447223 | | down |  |
| RGPD6 | | -1.4436327 | | -0.5297038 | | 1.4436327 | | down |  |
| CYP20A1 | | -1.4360507 | | -0.52210665 | | 1.4360507 | | down |  |
| SOCS7 | | -1.4279479 | | -0.5139434 | | 1.4279479 | | down |  |
| MCEE | | -1.42199 | | -0.5079114 | | 1.42199 | | down |  |
| KRTAP19-1 | | -1.4211948 | | -0.5071043 | | 1.4211948 | | down |  |
| SPTBN5 | | -1.4188392 | | -0.5047111 | | 1.4188392 | | down |  |
| IL15 | | -1.404906 | | -0.4904736 | | 1.404906 | | down |  |
| TRIQK | | -1.4042076 | | -0.4897562 | | 1.4042076 | | down |  |
| HCFC1 | | -1.4028215 | | -0.4883315 | | 1.4028215 | | down |  |
| SLC51B | | -1.4006948 | | -0.48614264 | | 1.4006948 | | down |  |
| GALNT13 | | -1.3919463 | | -0.47710356 | | 1.3919463 | | down |  |
| MLIP | | -1.3830849 | | -0.46788973 | | 1.3830849 | | down |  |
| RAD17 | | -1.3793124 | | -0.4639492 | | 1.3793124 | | down |  |
| BNIP3L | | -1.3763747 | | -0.4608733 | | 1.3763747 | | down |  |
| CDC25C | | -1.3696543 | | -0.45381176 | | 1.3696543 | | down |  |
| NRDE2 | | -1.366594 | | -0.4505846 | | 1.366594 | | down |  |
| ARHGAP18 | | -1.3653189 | | -0.4492379 | | 1.3653189 | | down |  |
| ZBTB1 | | -1.3640593 | | -0.4479064 | | 1.3640593 | | down |  |
| MTERF1 | | -1.3640445 | | -0.4478907 | | 1.3640445 | | down |  |
| RBPJ | | -1.3635294 | | -0.4473459 | | 1.3635294 | | down |  |
| EGLN3 | | -1.3605523 | | -0.44419244 | | 1.3605523 | | down |  |
| MCCC2 | | -1.3594198 | | -0.4429911 | | 1.3594198 | | down |  |
| FBXO11 | | -1.3589343 | | -0.44247565 | | 1.3589343 | | down |  |
| ITFG1 | | -1.3558819 | | -0.43923154 | | 1.3558819 | | down |  |
| ZNF660 | | -1.349958 | | -0.4329145 | | 1.349958 | | down |  |
| IL18 | | -1.3499018 | | -0.4328545 | | 1.3499018 | | down |  |
| ANKS1B | | -1.3482902 | | -0.43113104 | | 1.3482902 | | down |  |
| CLRN3 | | -1.3429437 | | -0.42539883 | | 1.3429437 | | down |  |
| DEPDC5 | | -1.3403219 | | -0.42257947 | | 1.3403219 | | down |  |
| C4BPB | | -1.3396528 | | -0.42185912 | | 1.3396528 | | down |  |
| ARPP19 | | -1.3383236 | | -0.420427 | | 1.3383236 | | down |  |
| ZNF440 | | -1.3377907 | | -0.4198525 | | 1.3377907 | | down |  |
| ZDHHC23 | | -1.3371072 | | -0.41911516 | | 1.3371072 | | down |  |
| EXO1 | | -1.3368418 | | -0.41882882 | | 1.3368418 | | down |  |
| ADORA2A | | -1.3368385 | | -0.41882515 | | 1.3368385 | | down |  |
| GTDC1 | | -1.3355409 | | -0.41742414 | | 1.3355409 | | down |  |
| INTS7 | | -1.334448 | | -0.41624308 | | 1.334448 | | down |  |
| FBLN5 | | -1.3304024 | | -0.4118627 | | 1.3304024 | | down |  |
| FANCM | | -1.3293569 | | -0.4107285 | | 1.3293569 | | down |  |
| CCDC180 | | -1.3278527 | | -0.4090952 | | 1.3278527 | | down |  |
| ARID4B | | -1.3278266 | | -0.40906683 | | 1.3278266 | | down |  |
| SETMAR | | -1.3230807 | | -0.40390104 | | 1.3230807 | | down |  |
| REV3L | | -1.3213046 | | -0.40196306 | | 1.3213046 | | down |  |
| FOXRED1 | | -1.3168771 | | -0.3971208 | | 1.3168771 | | down |  |
| EIF2AK4 | | -1.3148482 | | -0.3948962 | | 1.3148482 | | down |  |
| GLRB | | -1.3147792 | | -0.39482045 | | 1.3147792 | | down |  |
| MAP4K5 | | -1.3135456 | | -0.3934663 | | 1.3135456 | | down |  |
| SPX | | -1.3130394 | | -0.39291024 | | 1.3130394 | | down |  |
| RTTN | | -1.3090711 | | -0.38854343 | | 1.3090711 | | down |  |
| GLMN | | -1.3089986 | | -0.3884636 | | 1.3089986 | | down |  |
| VKORC1L1 | | -1.3076187 | | -0.3869419 | | 1.3076187 | | down |  |
| UNC93A | | -1.3061894 | | -0.38536412 | | 1.3061894 | | down |  |
| LEPR | | -1.3055 | | -0.38460246 | | 1.3055 | | down |  |
| WNK2 | | -1.3047196 | | -0.3837398 | | 1.3047196 | | down |  |
| CLINT1 | | -1.3013119 | | -0.37996674 | | 1.3013119 | | down |  |
| FAM111A | | -1.3011237 | | -0.37975818 | | 1.3011237 | | down |  |
| PLEKHH2 | | -1.297027 | | -0.37520853 | | 1.297027 | | down |  |
| ZNF124 | | -1.2874835 | | -0.36455393 | | 1.2874835 | | down |  |
| MTMR2 | | -1.2861507 | | -0.36305967 | | 1.2861507 | | down |  |
| ATP9B | | -1.2853483 | | -0.3621594 | | 1.2853483 | | down |  |
| HIRA | | -1.2807627 | | -0.35700312 | | 1.2807627 | | down |  |
| ZNF684 | | -1.2802454 | | -0.35642046 | | 1.2802454 | | down |  |
| THAP5 | | -1.2769071 | | -0.3526535 | | 1.2769071 | | down |  |
| CMTM4 | | -1.2764329 | | -0.35211772 | | 1.2764329 | | down |  |
| SLC4A4 | | -1.2745134 | | -0.34994647 | | 1.2745134 | | down |  |
| GYG2 | | -1.2742108 | | -0.34960395 | | 1.2742108 | | down |  |
| ARL16 | | -1.2724711 | | -0.34763288 | | 1.2724711 | | down |  |
| ZFHX4 | | -1.2710721 | | -0.3460459 | | 1.2710721 | | down |  |
| WDR18 | | -1.269253 | | -0.3439797 | | 1.269253 | | down |  |
| PTPN2 | | -1.26844 | | -0.34305525 | | 1.26844 | | down |  |
| DONSON | | -1.2672794 | | -0.34173456 | | 1.2672794 | | down |  |
| SMDT1 | | -1.2671815 | | -0.34162316 | | 1.2671815 | | down |  |
| USP33 | | -1.2665377 | | -0.34088993 | | 1.2665377 | | down |  |
| POLG2 | | -1.2653952 | | -0.339588 | | 1.2653952 | | down |  |
| FAM208B | | -1.2640843 | | -0.33809265 | | 1.2640843 | | down |  |
| SCAMP1 | | -1.2631425 | | -0.33701736 | | 1.2631425 | | down |  |
| ZNF827 | | -1.2625858 | | -0.33638138 | | 1.2625858 | | down |  |
| TMEM143 | | -1.2606221 | | -0.33413586 | | 1.2606221 | | down |  |
| SLC47A1 | | -1.2588067 | | -0.33205682 | | 1.2588067 | | down |  |
| CGGBP1 | | -1.2567831 | | -0.32973576 | | 1.2567831 | | down |  |
| ZNF552 | | -1.2552816 | | -0.32801104 | | 1.2552816 | | down |  |
| UTP14C | | -1.2552334 | | -0.32795563 | | 1.2552334 | | down |  |
| ZBTB37 | | -1.2534752 | | -0.32593343 | | 1.2534752 | | down |  |
| BHLHE40 | | -1.2515857 | | -0.32375717 | | 1.2515857 | | down |  |
| RGL4 | | -1.2503847 | | -0.32237196 | | 1.2503847 | | down |  |
| GALK2 | | 1.2500138 | | 0.32194406 | | 1.2500138 | | up |  |
| KIF5B | | 1.2508626 | | 0.32292336 | | 1.2508626 | | up |  |
| KTI12 | | 1.2571365 | | 0.3301413 | | 1.2571365 | | up |  |
| BTRC | | 1.2600859 | | 0.33352208 | | 1.2600859 | | up |  |
| ZNF554 | | 1.2688091 | | 0.34347504 | | 1.2688091 | | up |  |
| CRYL1 | | 1.2699547 | | 0.34477708 | | 1.2699547 | | up |  |
| SIRT2 | | 1.2705326 | | 0.3454334 | | 1.2705326 | | up |  |
| DAGLA | | 1.2746155 | | 0.3500622 | | 1.2746155 | | up |  |
| SPINK5 | | 1.2761292 | | 0.3517744 | | 1.2761292 | | up |  |
| RITA1 | | 1.2764318 | | 0.35211644 | | 1.2764318 | | up |  |
| WWTR1 | | 1.284383 | | 0.36107558 | | 1.284383 | | up |  |
| CLDN23 | | 1.2857709 | | 0.36263356 | | 1.2857709 | | up |  |
| MAP4K1 | | 1.2867446 | | 0.36372578 | | 1.2867446 | | up |  |
| TBCC | | 1.2889775 | | 0.36622715 | | 1.2889775 | | up |  |
| BCKDHB | | 1.2900753 | | 0.36745524 | | 1.2900753 | | up |  |
| PGGT1B | | 1.2918005 | | 0.36938334 | | 1.2918005 | | up |  |
| MICB | | 1.2943146 | | 0.37218833 | | 1.2943146 | | up |  |
| ACSS3 | | 1.2968543 | | 0.37501636 | | 1.2968543 | | up |  |
| EHHADH | | 1.2968647 | | 0.37502798 | | 1.2968647 | | up |  |
| DHRS13 | | 1.2973909 | | 0.3756132 | | 1.2973909 | | up |  |
| MTHFR | | 1.2981211 | | 0.37642503 | | 1.2981211 | | up |  |
| HIST1H2BJ | | 1.3044943 | | 0.38349056 | | 1.3044943 | | up |  |
| POLR3GL | | 1.3056442 | | 0.38476175 | | 1.3056442 | | up |  |
| SUMF1 | | 1.3059233 | | 0.38507023 | | 1.3059233 | | up |  |
| HEXB | | 1.3147762 | | 0.39481717 | | 1.3147762 | | up |  |
| ZNF629 | | 1.3175031 | | 0.39780635 | | 1.3175031 | | up |  |
| DCP1B | | 1.3233222 | | 0.40416437 | | 1.3233222 | | up |  |
| DHX30 | | 1.3273088 | | 0.408504 | | 1.3273088 | | up |  |
| BEND7 | | 1.3280554 | | 0.4093153 | | 1.3280554 | | up |  |
| DLX1 | | 1.3303752 | | 0.41183323 | | 1.3303752 | | up |  |
| SLC2A4 | | 1.3375932 | | 0.4196394 | | 1.3375932 | | up |  |
| FAM76A | | 1.3461044 | | 0.42879033 | | 1.3461044 | | up |  |
| SH3D21 | | 1.3462462 | | 0.42894232 | | 1.3462462 | | up |  |
| CAMKMT | | 1.3480436 | | 0.43086714 | | 1.3480436 | | up |  |
| HID1 | | 1.3486248 | | 0.43148905 | | 1.3486248 | | up |  |
| ZNF385A | | 1.3487087 | | 0.43157884 | | 1.3487087 | | up |  |
| RIN2 | | 1.3520062 | | 0.43510175 | | 1.3520062 | | up |  |
| AKNA | | 1.3527725 | | 0.43591926 | | 1.3527725 | | up |  |
| OLFM3 | | 1.3588336 | | 0.4423688 | | 1.3588336 | | up |  |
| C8A | | 1.369642 | | 0.45379886 | | 1.369642 | | up |  |
| HIST1H2AG | | 1.3696915 | | 0.45385098 | | 1.3696915 | | up |  |
| HCFC1R1 | | 1.3719187 | | 0.45619503 | | 1.3719187 | | up |  |
| CP | | 1.3734004 | | 0.45775238 | | 1.3734004 | | up |  |
| APOLD1 | | 1.3852307 | | 0.47012627 | | 1.3852307 | | up |  |
| ZNF619 | | 1.3946801 | | 0.47993428 | | 1.3946801 | | up |  |
| ARHGEF17 | | 1.4259255 | | 0.51189864 | | 1.4259255 | | up |  |
| HIST1H3H | | 1.4259397 | | 0.511913 | | 1.4259397 | | up |  |
| OGFOD2 | | 1.4348972 | | 0.52094734 | | 1.4348972 | | up |  |
| GLIS2 | | 1.4598799 | | 0.5458497 | | 1.4598799 | | up |  |
| SHQ1 | | 1.4604325 | | 0.54639566 | | 1.4604325 | | up |  |
| TMEM206 | | 1.4632306 | | 0.54915714 | | 1.4632306 | | up |  |
| OAZ3 | | 1.465335 | | 0.55123055 | | 1.465335 | | up |  |
| EVI5L | | 1.4781353 | | 0.5637784 | | 1.4781353 | | up |  |
| ZNF226 | | 1.4863169 | | 0.57174176 | | 1.4863169 | | up |  |
| TECPR2 | | 1.4940976 | | 0.5792744 | | 1.4940976 | | up |  |
| SH3KBP1 | | 1.5388453 | | 0.6218482 | | 1.5388453 | | up |  |
| SHH | | 1.5393263 | | 0.6222991 | | 1.5393263 | | up |  |
| NLRP2 | | 1.576016 | | 0.6562822 | | 1.576016 | | up |  |
| DBP | | 1.5839678 | | 0.663543 | | 1.5839678 | | up |  |
| DTD1 | | 1.588437 | | 0.6676078 | | 1.588437 | | up |  |
| KLRG1 | | 1.6393563 | | 0.7131294 | | 1.6393563 | | up |  |
| MCOLN1 | | 1.6501784 | | 0.72262204 | | 1.6501784 | | up |  |
| ICAM2 | | 1.6551515 | | 0.7269632 | | 1.6551515 | | up |  |
| NOTCH3 | | 1.6629114 | | 0.7337113 | | 1.6629114 | | up |  |
| SPARC | | 1.6676493 | | 0.73781586 | | 1.6676493 | | up |  |
| GSTM2 | | 1.6898398 | | 0.7568865 | | 1.6898398 | | up |  |
| MYL5 | | 1.7509203 | | 0.80811346 | | 1.7509203 | | up |  |
| PDGFD | | 1.8302268 | | 0.87202245 | | 1.8302268 | | up |  |
| COPZ2 | | 1.842433 | | 0.8816121 | | 1.842433 | | up |  |
| FAM86B1 | | 1.8905972 | | 0.9188421 | | 1.8905972 | | up |  |
| LDHB | | 1.9231639 | | 0.94348174 | | 1.9231639 | | up |  |
| MRPL23 | | 1.924441 | | 0.9444394 | | 1.924441 | | up |  |
| IL9R | | 1.9914476 | | 0.9938175 | | 1.9914476 | | up |  |
| RAB4B | | 2.0525107 | | 1.0373898 | | 2.0525107 | | up |  |
| DNAJC18 | | 2.2556908 | | 1.1735693 | | 2.2556908 | | up |  |
| HIST2H4A | | 2.6934216 | | 1.42944 | | 2.6934216 | | up |  |
| XCL2 | | 2.7364075 | | 1.4522831 | | 2.7364075 | | up |  |
| SNCA | | 3.0885408 | | 1.6269253 | | 3.0885408 | | up |  |
