# Supplementary figures and images for: CCR5 receptor antagonism inhibits hepatitis C virus (HCV) replication in vitro
Source: PLoS One. 2019 Oct 29;14(10):e0224523. doi: 10.1371/journal.pone.0224523 (PMC6818973; doi:10.1371/journal.pone.0224523)

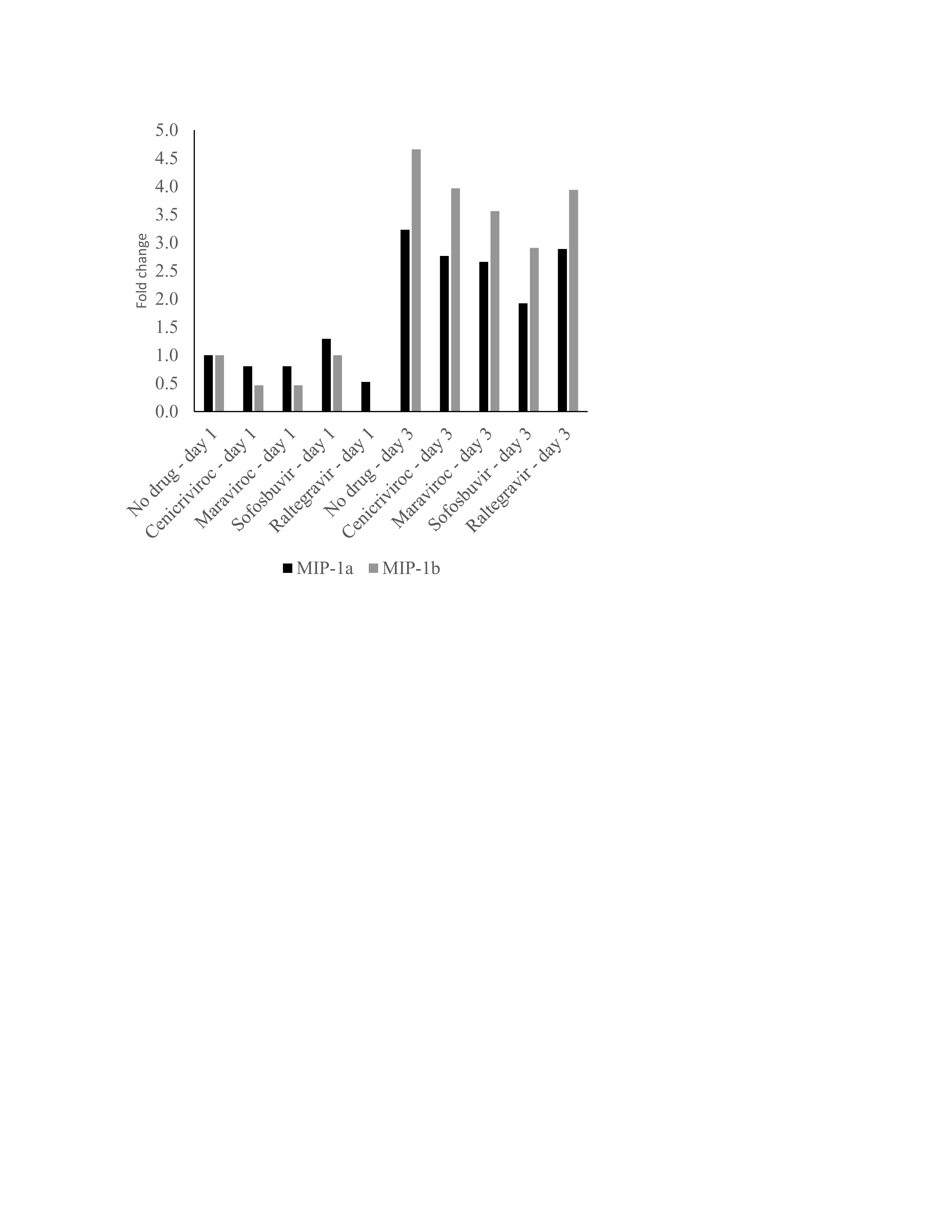

Supplement: S1 Fig — Expressed as fold change from day 1 no-drug control condition. (TIF) [file pone.0224523.s002.tif]
