# Supplementary material for: CCR5 receptor antagonism inhibits hepatitis C virus (HCV) replication in vitro
Source: PLoS One. 2019 Oct 29;14(10):e0224523. doi: 10.1371/journal.pone.0224523 (PMC6818973; doi:10.1371/journal.pone.0224523)
Supplement: S2 Fig — Original annotated gel image for Fig 2A. (PDF) [file pone.0224523.s003.pdf]

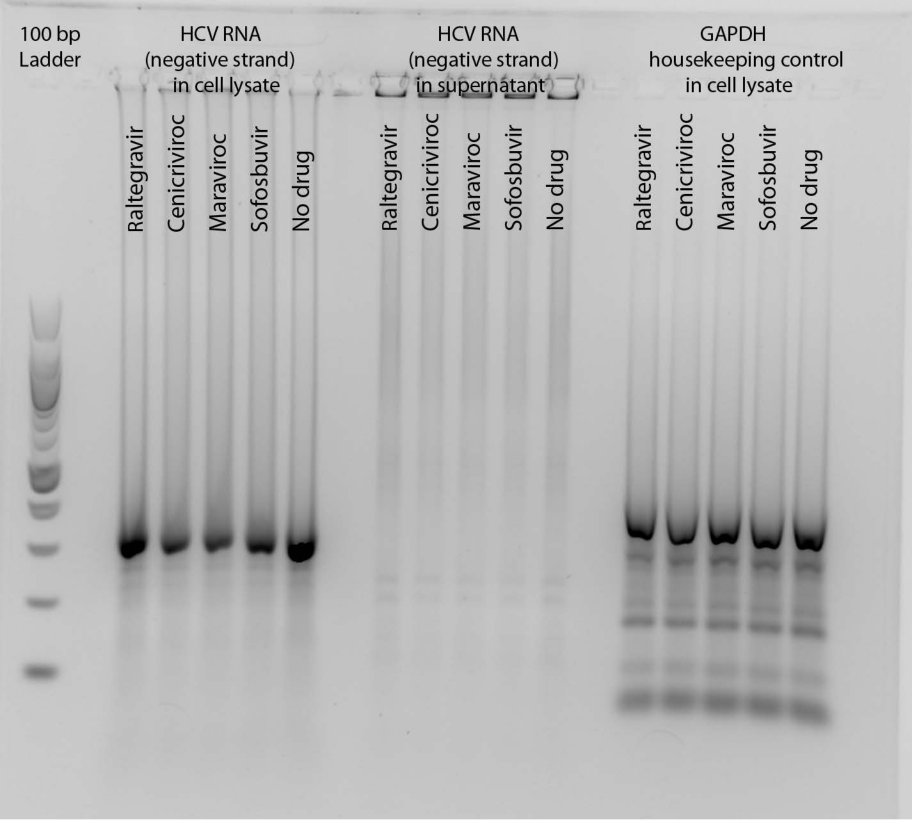
100 bp Ladder
HCV RNA (negative strand) in cell lysate
HCV RNA (negative strand) in supernatant
GAPDH housekeeping control in cell lysate
Raltegravir
Cenicriviroc
Maraviroc
Sofosbuvir
No drug
Raltegravir
Cenicriviroc
Maraviroc
Sofosbuvir
No drug
Raltegravir
Cenicriviroc
Maraviroc
Sofosbuvir
No drug
